# Comparative Assessment of Rapid Identification and Antimicrobial Susceptibility Testing Methods for Bloodstream Infections in a Non-24/7 Clinical Microbiology Laboratory

**DOI:** 10.3390/microorganisms13051041

**Published:** 2025-04-30

**Authors:** Sunggyun Park, Dohoon Kim, Namhee Ryoo

**Affiliations:** Department of Laboratory Medicine, Keimyung University School of Medicine, Daegu 42601, Republic of Korea; nosdolu2@gmail.com (S.P.); kdh@dsmc.or.kr (D.K.)

**Keywords:** bloodstream infection, antimicrobial susceptibility testing, turnaround time, non-24/7 microbiology laboratory

## Abstract

Rapid identification and antimicrobial susceptibility testing are essential for timely bloodstream infection (BSI) management. This study aimed to investigate the performance and turnaround time of multiple rapid diagnostic methods in a microbiology laboratory without 24/7 operation. This study included 236 positive blood culture bottles. Rapid identification methods were assessed with the SepsiTyper kit and the FilmArray blood culture identification 2 (BCID2) panel. Rapid antimicrobial susceptibility testing (AST) methods involved direct AST using the BD Phoenix M50 system and QuantaMatrix direct and rapid antimicrobial susceptibility testing (dRAST) and resistance gene detection with the FilmArray BCID2 panel. Conventional methods were used to compare results. The turnaround time was analyzed from blood culture positivity to preparation initiation and from preparation initiation to result reporting. Both rapid identification methods significantly reduced the turnaround time (~1 day and 19 h) compared to conventional identification. SepsiTyper demonstrated higher species-level accuracy in monomicrobial samples, whereas BCID2 outperformed in polymicrobial cases. Among the rapid AST methods, BCID2 and dRAST enabled result reporting within 24 h of positivity. Preparation delays were >45% of the overall turnaround time. Rapid diagnostics substantially shortened the BSI diagnostic time, even in limited-operation settings. Their clinical utility may be improved through 24/7 laboratory workflows.

## 1. Introduction

Bloodstream infection (BSI) is a significant global public health issue and is associated with considerable morbidity and mortality [1,2,3]. In 2013, BSI demonstrated an incidence of 113 and 204 cases per 100,000 population in North America and Europe, respectively [4]. A more recent population-based study from Finland (2004–2018) reported that the annual incidence of BSIs increased from 150 to 309 per 100,000 population, with a stable 30-day case fatality rate of approximately 13% [5]. In England, surveillance data from 2022–2023 showed an incidence of about 150 per 100,000 population for major BSI pathogens, with a 30-day case fatality rate of 17.6% [6]. Meanwhile, the global case fatality rate for BSI has been estimated to range from approximately 15–30% [3,4,5,6,7,8]. Kumar et al. previously revealed that inappropriate antimicrobial therapy reduced the survival rate of patients experiencing septic shock to one-fifth [9]. Furthermore, they demonstrated that each 1 h delay in effective antimicrobial therapy initiation after the onset of hypotension increased mortality by approximately 7.6% [10].

Considering these results, rapid and appropriate antimicrobial treatment is crucial for improving BSI-related mortality. However, variations in the distribution of causative pathogens and antimicrobial susceptibility patterns across different regions and periods increase the risk of errors in empirical antibiotic therapy [3]. The increasing prevalence of multidrug-resistant organisms further emphasizes the need for rapid and accurate diagnosis. Traditional blood culture methods require at least 48 h to determine pathogens and antimicrobial susceptibility, thereby posing challenges to the timely selection of effective antimicrobial therapy [11,12].

In recent years, advancements in technologies, such as mass spectrometry (MS), molecular diagnostics, and digital imaging, have brought significant changes to clinical microbiology laboratories [11,13]. Commercially available rapid identification tests for positive blood cultures include methods that directly process the blood culture broth without subculturing on solid media to collect a bacterial pellet, which is then determined using matrix-assisted laser desorption ionization time-of-flight (MALDI-TOF) MS (e.g., SepsiTyper kit [Bruker Daltonics, Bremen, Germany], SepsiPrep kit [ASTA Corp., Suwon, Republic of Korea]). Another approach involves molecular genetic methods that utilize pathogen-specific primers to directly detect key pathogens from positive blood culture bottles (e.g., FilmArray blood culture identification 2 [BCID2] panel [bioMérieux, Marcy-l’Étoile, France]) [13].

Rapid antimicrobial susceptibility testing (AST) from positive blood cultures is conducted with the several following methods: (1) direct inoculation of positive blood culture broth onto Mueller–Hinton agar followed by disk diffusion testing (e.g., direct blood culture disk diffusion testing of Clinical and Laboratory Standards Institute [CLSI], rapid AST of European Committee on Antimicrobial Susceptibility Testing); (2) processing the broth to obtain a bacterial pellet for determining the minimum inhibitory concentration (MIC) using conventional automated AST systems (e.g., direct AST using BD Phoenix M50 [Becton-Dickinson and Company, Sparks, NV, USA]); (3) utilizing microscopy-based imaging systems to detect morphological changes in bacteria under antimicrobial exposure, thereby assessing bacterial growth in real-time (e.g., Accelerate Pheno system [Accelerate Diagnostics, Tucson, AZ, USA], QuantaMatrix dRAST [QuantaMatrix, Seoul, Republic of Korea]); and (4) molecular methods that detect antimicrobial resistance (AMR) genes using gene-specific primers directly from positive blood culture bottles (e.g., FilmArray BCID2 panel) [11].

To translate the benefits of these rapid tests into improved patient care, a 24/7 laboratory operation and a timely reporting system are crucial [14]. However, not all laboratories can operate around the clock; thus, the potential advantages of rapid diagnostics are not being fully realized in practice [15,16]. Therefore, the benefits of rapid testing need to be evaluated in the context of each laboratory’s unique operational characteristics.

This study aimed to investigate the performance and practical use of various rapid identification and AST for blood cultures in a clinical microbiology laboratory with no 24/7 operations. We compared the results of the rapid identification tests currently accessible in Korea—SepsiTyper kit and FilmArray BCID2 panel—and rapid AST—direct AST using the BD Phoenix M50 (dPhoenix) system and QuantaMatrix direct and rapid AST (QdRAST) system—with those of the conventional methods.

## 2. Materials and Methods

### 2.1. Specimens

From early January to mid-June 2024, rapid identification and ASTs were conducted on 236 positive blood culture samples in which pathogens other than yeast or Gram-positive bacilli were identified by Gram staining. The Institutional Review Board of Keimyung University Dongsan Medical Center approved this study (DSMC IRB 2024-01-020), which adhered to the Declaration of Helsinki principles. The requirement for informed consent was waived as the study used residual blood culture broths obtained from routine clinical testing.

Our laboratory does not operate 24/7; however, results for both positive and negative blood cultures are reported around the clock, 7 days a week, using the Bact/ALERT VIRTUO Microbial Detection System (bioMérieux, Marcy l’Étoile, France). Conventional identification and AST, which includes positive blood culture subculturing, is performed daily from 8:30 a.m. to 5:30 p.m., 7 days a week. However, Gram staining of positive blood cultures is performed and reported twice daily (10:00–11:00 a.m. and 3:00–4:00 p.m. on weekdays only (Monday to Friday). Hence, this study included only positive blood culture samples processed on weekdays when Gram staining was conducted.

The samples for a more detailed analysis were categorized into two groups: Gram-positive cocci (119 samples) and Gram-negative bacilli (118 samples). Each group was subcategorized into a monomicrobial group, in which only one pathogen was determined based on Gram stain results, and a polymicrobial group, in which two or more pathogens were detected. This study demonstrated no polymicrobial samples containing both Gram-positive cocci and Gram-negative bacilli.

### 2.2. Conventional Identification and AST

In this study, all 236 positive blood culture bottles (Bact/ALERT FA/FN Plus; bioMérieux, Marcy l’Étoile, France) were subcultured onto blood agar plates after Gram staining. After overnight incubation at 35 °C, the isolated colonies were determined with the MALDI Biotyper Sirius system (Bruker Daltonics, Bremen, Germany). The acquired protein spectra were compared with the manufacturer’s reference library (v12), with identification scores of ≥2.000 indicated as species-level identification and scores between 1.800 and 1.999 denoted as genus-level identification.

AST for isolates identified as Gram-positive cocci was performed using the Vitek-2 system (bioMérieux, Marcy l’Étoile, France) with the AST-P601 card for *staphylococci*, AST-P600 card for *enterococci*, and AST-ST01 card for *streptococci*. Testing for Gram-negative bacilli was conducted using the NMIC-500 panel of the BD Phoenix M50 system (Becton, Dickinson and Company, Franklin Lakes, NJ, USA). The MIC results were interpreted into three clinical categories—susceptible, intermediate, and resistant—based on the CLSI M100 guidelines [17].

### 2.3. Rapid Identification

**Figure 1:**
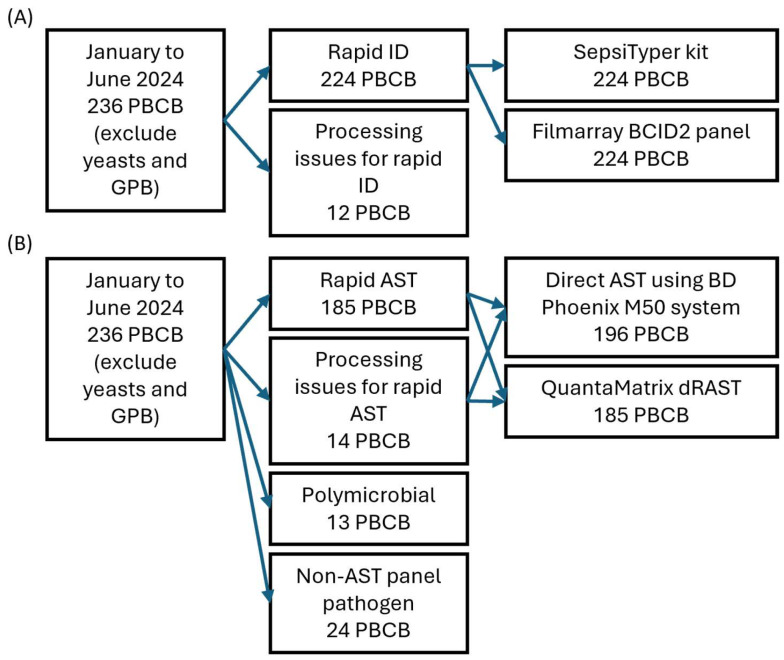
Study flowchart of rapid identification (**A**) and antimicrobial susceptibility tests (**B**). Due to processing issues and test availability, 196 PBCBs were tested with the BD Phoenix M50 system and 185 with QuantaMatrix dRAST. Abbreviations: PBCB, positive blood culture broth; GPB, Gram-positive bacilli; ID, identification; AST, antimicrobial susceptibility test.

Of the 236 samples included in this study, 12 were excluded from rapid identification due to test omission or reagent supply issues, including three samples not tested with the SepsiTyper kit, seven not tested with the BCID2 panel, and two not tested with either method. Hence, rapid identification using both the SepsiTyper kit and BCID2 panel was conducted on the remaining 224 positive blood culture samples (Figure 1A). All rapid identification tests were performed after confirming the presence of pathogens through Gram staining.

Sample preparation for rapid identification with the SepsiTyper kit for MALDI-TOF MS was performed following the standard SepsiTyper protocol, as described in previous reports [18]. In brief, positive blood culture broth at 1 mL was mixed with lysis buffer to lyse the cells, followed by two washing steps. Each lysis and washing step included centrifugation for 2 min—once during lysis and twice during washing. The resulting pellet was extracted using 70% formic acid and acetonitrile, and the supernatant was utilized for identification with the MALDI Biotyper Sirius system. The manufacturer estimates a hands-on time of approximately 30 min. The acquired protein spectra were compared with the manufacturer’s reference library (version 12), with identification scores of ≥1.800 interpreted as species-level identification and scores of 1.600–1.799 as genus-level identification.

BCID2 was performed following the manufacturer’s instructions. Briefly, positive blood culture broth at 0.2 mL was loaded into the pouch and inserted into the BioFire FilmArray 2.0 system (bioMérieux, Marcy l’Étoile, France). The manufacturer reports an estimated hands-on time of approximately 2 min. The panel utilized primer sets targeting 11 Gram-positive bacteria, 15 Gram-negative bacteria, and 7 yeasts to detect the presence of these organisms in the blood culture. Two BioFire FilmArray 2.0 systems, which were processed sequentially when more than two samples required testing, were used for analysis.

### 2.4. Rapid AST

Of the 236 samples included in this study, 13 polymicrobial samples identified by the conventional method and 24 samples in which the identified pathogens were not covered by the AST panels used (including 12 *Streptococcus* species, 3 *Micrococcus* species, 1 *Kytococcus* species, 1 *Janibacter* species, 1 *Haemophilus* species, 1 *Ochrobactrum* species, 1 *Achromobacter* species, 1 *Roseomonas* species, and 3 anaerobes) were excluded from rapid AST. Further, a total of 14 samples were excluded due to test omission, reagent supply issues, or instrument malfunction—of these, 11 were not tested with QdRAST only, and 3 were not tested with either method. Hence, both dPhoenix and QdRAST were performed on the remaining 185 samples (85 Gram-positive and 100 Gram-negative; Figure 1B). Among the 185 samples tested with both rapid AST methods, two could not be tested with the BCID2 panel due to reagent supply issues. All rapid AST procedures were initiated after confirming the presence of pathogens via Gram staining, following the same process as for rapid identification.

All dPhoenix experiments were conducted following the manufacturer’s instructions. Briefly, positive blood culture broth at 5 mL was transferred into a serum-separating tube with a 5 mL syringe. The tube was then centrifuged at 3000 rpm for 20 min. After discarding the supernatant, the collected bacteria in the gel layer were retrieved with a sterile swab. The swab was then suspended in identification broth (Becton-Dickinson and Company, Sparks, NV, USA) and adjusted to a 0.5 McFarland standard. Antimicrobial susceptibility testing was conducted using the PMIC-84 panel for Gram-positive cocci and the NMIC-500 panel for Gram-negative bacilli on the BD Phoenix M50 system.

The QdRAST system was used following the manufacturer’s instructions. In brief, positive blood culture broth at 1 mL was transferred into a sterile plain tube and loaded into the QdRAST system. The system then automatically performed the preprocessing steps, including mixing the sample with liquid agar, inoculating it into a microwell plate, and allowing the agar to solidify. These steps were automated; thus, the hands-on time was minimal. Antimicrobial susceptibility testing was conducted using the QuantaMatrix Gram-positive panel for Gram-positive cocci and the Gram-negative panel for Gram-negative bacilli. During the course of the study, the QdRAST software was upgraded, and analyses were conducted using both versions 1.5.1 and 1.6.1. Further, the results from both versions were compared.

BCID2 was performed as previously described. The panel detects the presence of AMR genes directly from positive blood culture bottles using primer sets targeting 10 AMR genes, including mecA/C, mecA/C with MREJ, vanA/B, CTX-M, IMP, KPC, mcr-1, NDM, OXA-48-like, and VIM.

### 2.5. Data Analysis and Statistics

A correct identification case for identification analysis in the monomicrobial group involved a case in which the results of rapid identification matched those of conventional identification at the species or genus level. A complete identification case in the polymicrobial group was defined as the correct identification at the species level of all microorganisms detected with conventional methods. A partial identification case was defined as the correct identification at the genus level or higher of at least one of the organisms determined using conventional methods. To reflect the practical utility of BCID2 in real-world clinical settings, organisms not detected due to absence from the target panel were classified as incorrect identifications, as they would result in an unreported pathogen in routine use despite being analytically undetectable. Further, identifications made at the genus level or higher due to the panel’s design (i.e., not targeting species level) were counted as correct at the genus level, not the species level. The McNemar chi-square test was conducted to compare the correct identification rates between the two rapid identification tests, and a *p*-value of <0.05 indicated statistical significance.

The turnaround time for identification was the interval from the time a blood culture signaled positive to the time the result of each identification test was reported. The turnaround time for conventional identification in our laboratory is not separately recorded, as the identification results are reported only after completing the corresponding AST. Therefore, the turnaround time for conventional identification was the same as that of conventional AST, measured from the time of blood culture positivity to the time of AST result reporting. To compare the paired turnaround times, the Friedman test was conducted, and a *p*-value of <0.05 indicated statistical significance.

Cases for AST analysis in which dPhoenix or QdRAST failed to yield results due to bacterial growth failure were defined as growth failure cases. The McNemar chi-square test was used to compare the growth failure rates of the two rapid AST methods, and a *p*-value of <0.05 indicated statistical significance.

Concordance with conventional AST was assessed for 166 samples (67 Gram-positive cocci and 99 Gram-negative bacilli) for which both dPhoenix and QdRAST were successfully performed and interpreted. Agreement was assessed following the CLSI M23 document, and the results were expressed as essential agreement (EA), categorical agreement (CA), very major error (VME), major error (ME), and minor error (mE) [19]. VME rates were calculated with the number of resistant isolates as the denominator, and ME rates were calculated with the number of susceptible isolates as the denominator. The McNemar chi-square test was conducted to compare CA rates between each rapid AST method and the conventional AST, and a *p*-value of <0.05 indicated statistical significance.

To clarify the clinical significance of detecting the 10 AMR genes for BCID2, each gene result was compared with the corresponding antimicrobial susceptibility phenotype. Specifically, mecA/C and MREJ detection were compared with the oxacillin MIC category for *Staphylococcus aureus* (SAU), and mecA/C detection alone was compared with the oxacillin MIC category for coagulase-negative *Staphylococci* (CNS). The detection of vanA/B was compared with the vancomycin MIC category for *Enterococcus* species. CTX-M detection was compared with both the cefepime MIC category and phenotypic extended-spectrum beta-lactamases (ESBL) test results, which were included in both dPhoenix and QdRAST for *Enterobacterales*. Carbapenemase genes (IMP, KPC, NDM, OXA-48-like, and VIM) were compared with the meropenem MIC category for *Enterobacterales*.

The turnaround time for ASTs was the time from blood culture positivity to AST result reporting. Unlike the identification tests, the ASTs in this study involved more complex preparation steps and included multiple testing methods. Hence, differences were found in the time taken to initiate preparation for each test. To minimize the effect of these differences on the overall turnaround time, the overall turnaround time was subcategorized into the time from blood culture positivity to test preparation initiation and the time from preparation initiation to result reporting. This approach enabled a more accurate estimation and comparison of the overall turnaround times across different AST methods. The Friedman test was conducted to compare the paired turnaround times, and a *p*-value of <0.05 indicated statistical significance. R (version 4.3.1; R Foundation for Statistical Computing, Vienna, Austria) was used for all statistical analyses.

## 3. Results

### 3.1. Accuracy of the Rapid Identification Methods

In monomicrobial Gram-positive cocci, the species-level correct identification rate was significantly higher in the SepsiTyper kit at 88.35% (95% confidence interval [CI]: 80.53–93.83%) compared to 64.08% (95% CI: 54.03–73.30%) in the BCID2 panel (Table 1). However, no significant difference was found between the two methods in genus-level correct identification rates.

In Gram-negative bacilli, the SepsiTyper kit demonstrated a higher species-level correct identification rate at 96.30% (95% CI: 90.79–98.98%) compared to the BCID2 panel at 88.89% (95% CI: 81.40–94.13%). At the genus level, the SepsiTyper kit exhibited superior accuracy, with a correct identification rate of 97.22% (95% CI: 90.79–98.98%), compared to the BCID2 panel at 89.81% (95% CI: 82.51–94.80%).

In polymicrobial Gram-positive cocci, the complete identification rate was 20% (95% CI: 2.52–55.61%) with the SepsiTyper kit and 40% (95% CI: 12.16–73.76%) with the BCID2 panel. The BCID2 panel demonstrated higher accuracy, but the difference was not statistically significant due to the small number of polymicrobial samples (Table 2).

Similarly, in polymicrobial Gram-negative bacilli, the complete identification rate was 0% (95% CI: 0–70.76%) with the SepsiTyper kit and 66.67% (95% CI: 9.43–99.16%) with the BCID2 panel, with the BCID2 panel demonstrating higher accuracy, although without statistical significance. Both rapid identification methods achieved 100% accuracy at the partial identification level.

Among monomicrobial isolates, the leading cause for incorrect species-level identification with the SepsiTyper kit was a low MALDI-TOF MS identification score resulting in genus-level identification, accounting for 56.25% (9/16) of cases. The second common cause was the complete failure to determine the pathogen due to a low identification score, observed in 25% (4/16) of cases (Table S1).

The leading cause for correct species-level identification for the BCID2 panel was the determination at the higher genus level, as the panel does not target species-level resolution, accounting for 65.31% (32/49) of cases. The second predominant cause, accounting for 18.37% (9/49) of cases, was the absence of the conventional method-identified pathogen from the panel’s target list, resulting in nondetection. Notably, in 8.16% (4/49) of cases, the BCID2 panel detected an additional pathogen that was not found by the conventional method.

Among the polymicrobial isolates with incorrect complete-level identification, the SepsiTyper kit failed to detect one of the two pathogens in 100% (4/4) of cases (Table S2). In the BCID2 panel, 100% (7/7) of cases with incorrect complete-level identification were due to one or more pathogens being identified only at the genus level, as the panel did not target those organisms at the species level.

### 3.2. Turnaround Time of the Rapid Identification Methods

The median turnaround time from blood culture positivity to reporting of pathogen identification with the SepsiTyper kit was approximately 9 h 56 min for Gram-positive cocci and 11 h 9 min for Gram-negative bacilli (Table 3). The median turnaround times for the BCID2 were approximately 9 h 59 min for Gram-positive cocci and 9 h 33 min for Gram-negative bacilli. The Friedman test revealed no statistically significant difference in the turnaround times between the two rapid identification methods (Table 4).

**Figure 2:**
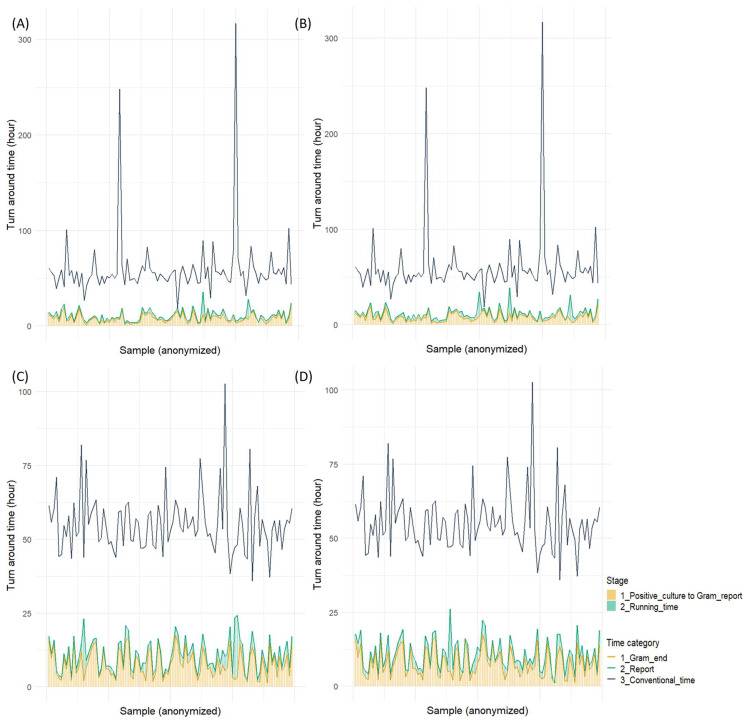
Turnaround time for two rapid identification tests: (**A**) SepsiTyper kit for Gram-positive cocci, (**B**) Filmarray BCID2 panel for Gram-positive cocci, (**C**) SepsiTyper kit for Gram-negative bacilli, (**D**) Filmarray BCID2 panel for Gram-negative bacilli.

However, both rapid identification methods demonstrated statistically significant reductions in turnaround time compared to the conventional method, with a median time saving of >1 day and 19 h. The median turnaround time of Gram staining was approximately 7 h, accounting for approximately 70% of the overall turnaround time of both rapid identification methods (Figure 2).

### 3.3. Agreement Analysis of the Rapid AST Methods

Frequency analysis was conducted for growth failure cases, defined as failure to yield AST results due to bacterial growth failure within the instrument, among 196 samples tested with dPhoenix and 185 samples tested with QdRAST. The growth failure rates in the Gram-positive cocci were 16.85% (15/89) with dPhoenix and 8.24% (7/85) with QdRAST, with no statistically significant difference between them (*p* = 0.0614). The growth failure rates in the Gram-negative bacilli were 0.93% (1/107) with dPhoenix and 0% (0/100) with QdRAST. Further review of the conventional identification of Gram-positive cocci samples with growth failure revealed that 86.67% (13/15) of dPhoenix failures were CNS, with *Staphylococcus epidermidis* accounting for 73.33% (11/15). Similarly, among the QdRAST failures, 85.71% (6/7) were CNS, whereas *Staphylococcus epidermidis* accounted for 28.57% (2/7).

Results from both rapid AST methods were available and analyzed for 18 SAU, 28 CNS, 21 Enterococcus species, 83 Enterobacterales, and 16 non-fermentative Gram-negative bacilli (NFGNB) isolates (Tables S3 and S4).

The CA rates for SAU, CNS, Enterococcus, Enterobacterales, and NFGNB species were 98.89%, 91.03%, 96.43%, 95.47%, and 96.64% with dPhoenix and 95.56%, 88.34%, 88.10%, 90.94%, and 86.58% with QdRAST version 1.5.1, respectively. Among the Gram-positive cocci included in this study, the lowest CA was observed for the CNS–gentamicin combination, accounting for 57.14% with dPhoenix and 53.57% with QdRAST. No VMEs were observed for this combination in either rapid AST method. Combinations for which the CA of QdRAST version 1.5.1 was lower than that of the dPhoenix included SAU–penicillin, CNS–erythromycin, and Enterococcus–linezolid, with MEs or mEs being the leading cause of discrepancies. VMEs for the CNS were observed in both rapid AST methods, accounting for 7.02% (4/57) with dPhoenix and 5.26% (3/57) with QdRAST. However, no statistically significant differences in CA were observed between the two rapid AST methods for any Gram-positive cocci–antimicrobial agent combination.

The CA rates for SAU, CNS, and Enterococcus species using QdRAST version 1.6.1 were 95.29%, 90.14%, and 92.86%, respectively, demonstrating improvement compared with version 1.5.1. Notably, all three previously observed VMEs in the CNS with version 1.5.1 were resolved and no longer present in version 1.6.1. The CA rates improved in previously underperforming combinations, such as SAU–penicillin, CNS–erythromycin, and Enterococcus–linezolid, with these combinations demonstrating lower CA rates compared with dPhoenix.

The CA rates for Enterobacterales and NFGNB were 95.47% and 96.64% with dPhoenix and 90.94% and 86.58% with QdRAST version 1.5.1, respectively. The combinations in which QdRAST version 1.5.1 demonstrated lower CA than dPhoenix included Enterobacterales–ampicillin/sulbactam, Enterobacterales–piperacillin/tazobactam, NFGNB–amikacin, and NFGNB–ciprofloxacin. Among these, Enterobacterales–ampicillin/sulbactam (*p* = 0.0059) and Enterobacterales–piperacillin/tazobactam (*p* = 0.0412) exhibited statistically significant differences. Most of the lower CA rates observed with QdRAST version 1.5.1 were due to MEs or mEs, whereas the Enterobacterales–piperacillin/tazobactam combination demonstrated a notably high rate at 50% (six isolates). Further, a similarly high VME rate of 41.57% (five isolates) was observed with dPhoenix for this same combination.

The CA rates for Enterobacterales and NFGNB improved to 92.63% and 89.05% with QdRAST version 1.6.1, respectively. In particular, 10 MEs and 7 mEs previously observed in Enterobacterales were corrected, and 2 VMEs in NFGNB were resolved. Furthermore, combinations that demonstrated statistically significant CA rate differences in version 1.5.1, including Enterobacterales–ampicillin/sulbactam and Enterobacterales–piperacillin/tazobactam, no longer exhibited significant differences in version 1.6.1. However, the high VME rate for Enterobacterales–piperacillin/tazobactam observed in version 1.5.1 remained unresolved in version 1.6.1.

Table 5 summarizes the predictive performance of the BCID2 panel for the conventional AST results. The panel accurately predicted both resistance and susceptibility (100% accuracy) for the SAU–oxacillin, Enterococcus–vancomycin, and Enterobacterales–meropenem combinations. The ability to predict cefepime susceptibility and ESBL phenotype for Enterobacterales showed a sensitivity of >80% and specificity of >90%. However, the predictive performance for oxacillin susceptibility in the CNS was limited, with a sensitivity of only 25%.

### 3.4. Turnaround Time of Rapid AST Methods

To account for differences in the time elapsed between blood culture positivity and the start of test preparation, the turnaround times for dPhoenix, both versions of QdRAST, BCID2, and conventional AST were analyzed by categorizing the turnaround time from blood culture positivity to test preparation initiation and from preparation initiation to result reporting. Table 6 and Table S5 present the detailed results of this analysis.

The median time from blood culture positivity to preparation initiation for each rapid AST method for Gram-positive cocci and Gram-negative bacilli was approximately 12 h 47 min and 12 h 14 min with dPhoenix, 11 h 52 min and 11 h 51 min with QdRAST, and 8 h 29 min and 8 h 52 min with the BCID2 panel, respectively. QdRAST began preparation approximately 15 min earlier than dPhoenix, whereas BCID2 began preparation approximately 3 h earlier than both methods. These differences were statistically significant (Table S5). The analysis of the time from blood culture positivity to preparation initiation for the BCID2, which is the shortest among all rapid AST methods, according to on-duty versus off-duty hours, revealed a median of approximately 4 h 19 min and 10 h 19 min, respectively (Figure S1).

Excluding the time from blood culture positivity to preparation initiation, the median time from preparation initiation to AST result reporting for Gram-positive cocci and Gram-negative bacilli was approximately 15 h 37 min and 13 h 57 min with dPhoenix (Table 6), 6 h 45 min and 6 h 43 min with QdRAST version 1.5.1, and 6 h 44 min and 6 h 39 min with QdRAST version 1.6.1, respectively. BCID2 demonstrated the shortest time from preparation initiation to result reporting for both Gram-positive cocci and Gram-negative bacilli, with a median of approximately 1 h 31 min. QdRAST version 1.6.1 exhibited up to a 2 h reduction in time for Gram-negative bacilli compared to QdRAST version 1.5.1 but with no statistically significant difference (Table S5). However, both versions of QdRAST reduced the time by more than 8 h and 30 min compared to dPhoenix (Table S5). The BCID2 reduced the time by approximately 14 h 21 min for Gram-positive cocci and 12 h 33 min for Gram-negative bacilli compared to dPhoenix. Further, it outperformed QdRAST by approximately 5 h and 10 min in both Gram-positive cocci and Gram-negative bacilli.

**Figure 3:**
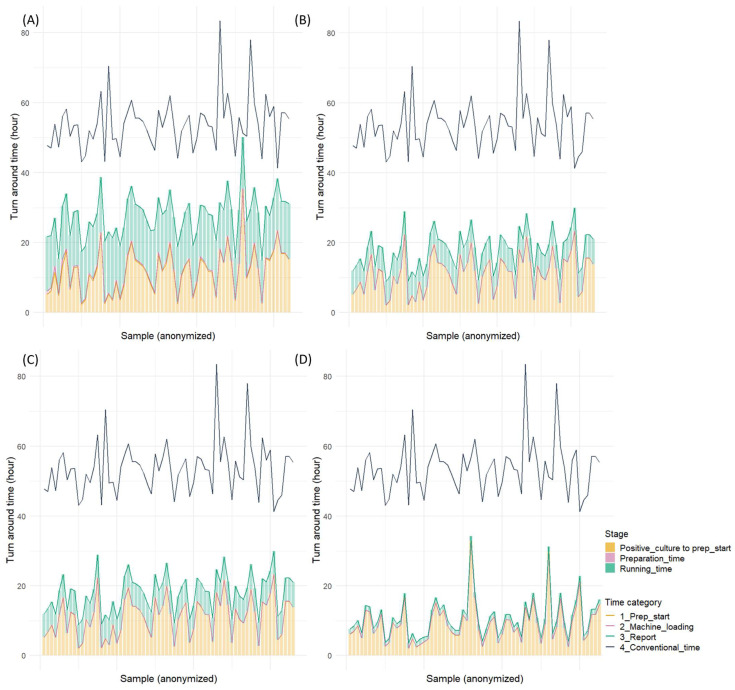
Turnaround time for four rapid antimicrobial susceptibility tests (Gram-positive cocci): (**A**) direct AST using BD Phoenix M50 system, (**B**) QuantaMatrix dRAST 1.5.1 version, (**C**) QuantaMatrix dRAST 1.6.1 version, and (**D**) Filmarray BCID2 panel.

**Figure 4:**
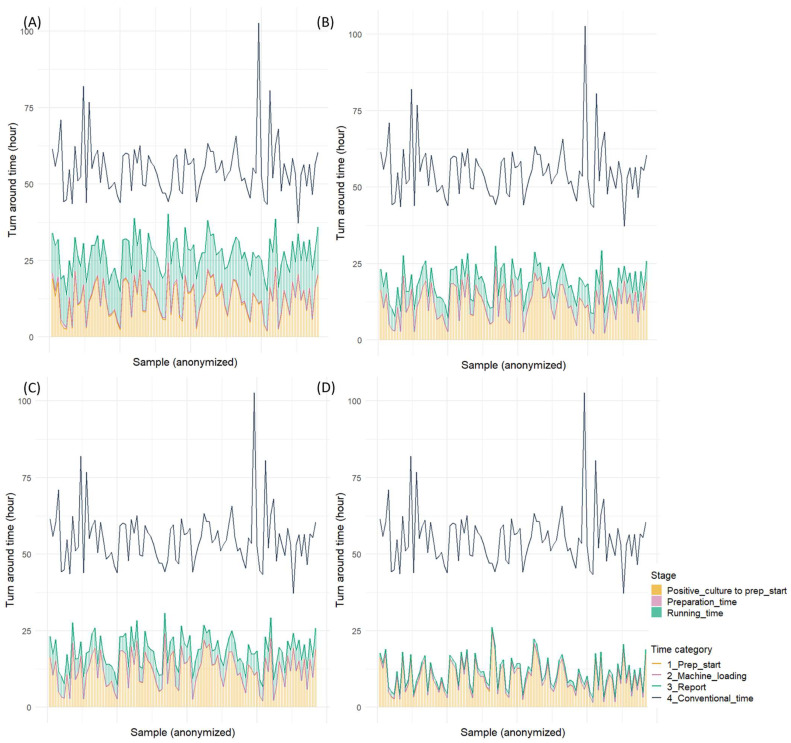
Turnaround time for four rapid antimicrobial susceptibility tests (Gram-negative bacilli): (**A**) direct AST using BD Phoenix M50 system, (**B**) QuantaMatrix dRAST 1.5.1 version, (**C**) QuantaMatrix dRAST 1.6.1 version, and (**D**) Filmarray BCID2 panel.

The comparison of the overall turnaround time from blood culture positivity to result reporting revealed that all rapid AST methods significantly reduced the time compared to the conventional method (Table S5). The BCID2, which demonstrated the shortest turnaround time among all methods, reduced the time by approximately 1 day and 19 h and achieved an overall turnaround time of approximately 10 h. QdRAST reduced the turnaround time by approximately 1 day and 11 h compared to the conventional method, with an overall turnaround time of approximately 18 h. Moreover, dPhoenix showed a reduction of approximately 1 day and 2 h, with an overall turnaround time of 1 day and 3 h. The analysis of the proportion of the time from blood culture positivity to preparation initiation revealed that this phase accounted for approximately 45% of the total time with dPhoenix, 63% with QdRAST, and >80% with BCID2 (Figure 3 and Figure 4).

## 4. Discussion

Several previous studies have indicated that the 24 h operation of clinical microbiology laboratories is crucial for the timely diagnosis and treatment of BSIs [15,16,20,21,22]. Delays in processing positive blood cultures during nighttime or weekends significantly prolong the time to pathogen identification and appropriate therapy initiation. Not all clinical microbiology laboratories operate 24/7. A European study revealed that only approximately 13% of laboratories provided around the clock services for the immediate processing of positive blood cultures [22]. A Korean report indicated that only 0.9% of institutions performed microbial identification or AST during weekday night shifts [15]. These results emphasize the need to assess the use of rapid identification and AST in laboratories with no 24/7 operations and underscore the importance of optimizing diagnostic strategies for BSI within limited-resource settings.

Most previous studies on rapid blood culture identification and AST have focused on comparing new rapid methods with conventional culture-based techniques, primarily investigating the accuracy of pathogen identification and turnaround time reduction [13,23,24,25,26,27,28,29,30,31,32,33,34,35,36,37,38,39,40,41,42,43,44]. These comparative studies have revealed that rapid diagnostic methods shorten the time to results by several hours or even days compared to conventional methods [11,13,45]. The present study is unique because it not only compares rapid identification and AST methods with conventional approaches but also conducts direct comparisons between different rapid testing platforms. This study aimed to objectively evaluate the contribution of each test in the context of a non-24/7 laboratory setting by assessing multiple rapid identification and AST methods simultaneously.

The SepsiTyper kit and BCID2 are rapid identification tests that directly determine pathogens from positive blood culture bottles. The SepsiTyper kit is a sample preparation tool designed for use with MALDI-TOF MS, enabling the protein profile analysis of pathogens directly from blood culture broth [13]. However, the protein profiles obtained from positive blood cultures are limited in quality; thus, the reported identification accuracy of the SepsiTyper kit is approximately 80%. Notably, lower identification rates have been found for Gram-positive bacteria and yeasts [13].

Unlike the SepsiTyper kit, BCID2 uses a molecular approach, performing multiplex polymerase chain reactions targeting predefined BSI pathogens and AMR genes. The entire process takes approximately 1 h [11]. Due to its principle of detecting amplified genetic material, the test provides high sensitivity and specificity, with multicenter studies reporting sensitivity and specificity rates of >99% [43].

This study revealed that the SepsiTyper kit in monomicrobial samples demonstrated a higher correct species-level identification rate than BCID2 when both were compared with conventional identification methods (Table 1). Previous studies on the performance of BCID2 have revealed correct overall identification rates of approximately 83–90% [35,39,41,43,46], which is congruent with the genus-level accuracy observed in this study. The relatively lower species-level accuracy of the BCID2 panel was primarily due to off-target results, accounting for >83% of the incorrect identifications. Among these, approximately 78% could still be determined at the genus level or higher (Table S1). These results indicate that the key limitation of the BCID2 panel as a rapid identification tool lies in its restricted target range. This emphasizes the inherent strength of untargeted approaches, such as MALDI-TOF MS, which are not limited by predefined pathogen panels.

The number of polymicrobial samples included in this study was limited; however, the complete-level correct identification rate was higher with the BCID2 panel than with the SepsiTyper kit, unlike in the monomicrobial cases (Table 2). Similarly, Smith et al. previously reported that the BCID2 panel achieved a complete identification rate of 92.9% in polymicrobial samples, markedly higher than the 10.7% observed with the SepsiTyper kit [46]. In this study, all incomplete identifications with the SepsiTyper kit in polymicrobial samples were due to the detection of only one of the multiple pathogens. Hence, the partial-level correct identification rate was 100%. This is consistent with earlier results, where the SepsiTyper kit demonstrated a complete identification rate of approximately 34.3% and a partial identification rate of 54.5% in polymicrobial samples [47]. MALDI-TOF MS-based identification is less accurate in polymicrobial samples, but these limitations may be overcome through continuous updates to the reference database and improvements in sample preparation protocols, indicating the potential for improved performance in complex specimens.

This study revealed that both the SepsiTyper kit and BCID2 reduced the turnaround time by over 1 day and 19 h compared to the conventional method even in a microbiology laboratory that does not operate 24/7 and performs Gram staining only twice daily. However, no statistically significant difference in turnaround time was observed between the two rapid identification methods. In both tests, the time taken to obtain Gram stain results accounted for >70% of the total turnaround time. Therefore, in laboratories with 24/7 operations, at least 70% of the total turnaround time is expected to be further reduced, emphasizing the added value of continuous laboratory operation in maximizing the benefit of rapid diagnostic methods.

Both dPhoenix and QdRAST are rapid AST methods that can be performed directly from positive blood culture bottles without requiring subculturing to obtain isolated colonies [11]. Further, dPhoenix uses an automated broth microdilution platform to perform AST directly from bacterial pellets collected from the positive blood culture broth. Previous studies have revealed CA rates of 95–99% compared to conventional AST methods for both Gram-positive cocci and Gram-negative bacilli, indicating a high concordance level with conventional testing methods [28,30,31,42,44]. However, similar to other growth-based AST methods, this approach requires sufficient time for bacterial growth in the presence of antibiotics to identify the MIC, which represents a fundamental limitation in terms of speed.

The QdRAST is an innovative system that uses time-lapse microscopic imaging technology to evaluate bacterial growth under antibiotic exposure [11]. Unlike traditional broth microdilution methods, which assess bacterial growth macroscopically, this approach enables earlier detection of growth inhibition, allowing faster antimicrobial susceptibility determination. However, image-based interpretation can be challenging for certain organisms or under specific conditions, thereby posing a limitation to its universal applicability. Hence, the system is currently validated for a limited range of organisms, including *Staphylococcus* species, *Enterococcus* species, *Enterobacterales*, and NFGNB. In previous studies, QdRAST has shown CA rates of approximately 90–95% compared with conventional AST methods for both Gram-positive cocci and Gram-negative bacilli [26,32,33,36,38].

Both rapid AST methods are performed using positive blood culture broth, indicating that the bacteria are concentrated directly from the broth, unlike conventional testing with isolated colonies. This enrichment process significantly affects the test performance; thus, samples with low bacterial recovery frequently cause test failures. In this study, we revealed a higher failure rate in testing Gram-positive cocci compared to Gram-negative bacilli. Specifically, the failure rate for Gram-positive cocci was higher with dPhoenix (16.85%) than with QdRAST (8.24%). These failures were particularly prominent in the CNS group. This result is consistent with previous studies. Lupetti et al. revealed a failure rate of approximately 15% with dPhoenix, with the majority of failures occurring in *Staphylococcus* species [42]. Similarly, Wong et al. demonstrated a growth failure rate of approximately 1% with QdRAST [48]. Considering the high failure rate observed in the CNS when using dPhoenix, further research is warranted to investigate whether protocol modifications, such as increasing the bacterial concentration during the enrichment step, could reduce test failures and improve the overall performance in this group.

In this study, the CA for Gram-positive cocci and Gram-negative bacilli was approximately 94.87% and 95.60% with dPhoenix, 90.97% and 90.44% with QdRAST version 1.5.1, and 92.45% and 92.24% with QdRAST version 1.6.1, respectively, indicating that the software upgrade contributed to improved performance in both organism groups.

Among the bacterial groups analyzed, statistically significant differences in the CA rate with the conventional method were observed for two bacterial group–antimicrobial agent combinations: *Enterobacterales*–ampicillin/sulbactam and *Enterobacterales*–piperacillin/tazobactam. In both combinations, dPhoenix demonstrated a significantly higher CA rate compared with QdRAST version 1.5.1 (Table S3). The CA for these two combinations improved after the software update of QdRAST version 1.6.1, and the statistically significant differences between the two rapid AST methods were no longer observed (Table S4). However, dPhoenix continued to demonstrate relatively higher CA rates in these combinations.

Both rapid AST methods for CNS showed low CA rates at approximately 50% for gentamicin susceptibility. This may be attributed to the diverse resistance mechanisms associated with gentamicin. Cayci et al. previously evaluated the performance of three AST methods for gentamicin and revealed that certain aminoglycoside-modifying enzymes affect test outcomes [49], and various such enzymes have been reported in *Staphylococcus* species [50]. The low CA in SAU observed for penicillin in QdRAST is congruent with the results of Huh et al. [37]. However, according to the CLSI M100 document [17], penicillin susceptibility in SAU should be confirmed by testing for beta-lactamase production, and penicillin is rarely used as a first-line therapy due to the high prevalence of penicillinase-producing strains, thereby limiting the clinical effect of this discrepancy. Previous reports have revealed similar findings for combinations, including CNS–erythromycin and *Enterococcus*–linezolid, where low CA rates were observed in this study [26,36]. Both erythromycin and linezolid are bacteriostatic agents; thus, the limited ability to fully assess antimicrobial effects in a short timeframe may explain the reduced agreement. All VMEs observed in Gram-positive cocci were resolved, and the overall CA rates improved after the software upgrade of QdRAST version 1.6.1. This emphasizes the importance of continuous software optimization for rapid tests, such as QdRAST, that depend on imaging-based growth analysis. However, the lack of improvement in certain combinations, such as CNS–gentamicin, and the persistently low CA rates in others even after the update indicate that further refinement remains necessary to improve the accuracy and reliability of QdRAST.

This study observed a notably high VME rate in Gram-negative bacilli, particularly for the piperacillin/tazobactam combination. This finding is consistent with previous reports that have documented high VME rates or low CA for this antimicrobial agent [32,33,48]. Moreover, even when using isolated colonies, some studies have revealed that AST results for piperacillin/tazobactam vary between different testing platforms [51]. Importantly, in the updated QdRAST version 1.6.1, a high VME rate persisted for the Enterobacterales–piperacillin/tazobactam combination. This emphasizes the need for cross-validation using more than one AST method, particularly for crucial drug–pathogen combinations, and highlights the importance of continued efforts to improve the testing accuracy for this antimicrobial agent.

The BCID2 panel, as a rapid AST tool, differs fundamentally from the two phenotypic rapid AST methods described earlier in that it does not assess bacterial growth in the presence of antibiotics [11]. Instead, it directly detects the presence or absence of AMR genes from positive blood culture bottles. This molecular approach provides immediate clues regarding potential resistance and is faster than phenotypic methods; however, it does not assess how the bacteria respond to antibiotics in vitro. Hence, BCID2 cannot detect resistance mechanisms that are not associated with the included AMR genes, and discrepancies may appear between the genotypic and phenotypic susceptibility results. Therefore, the panel cannot independently confirm susceptibility or resistance, and additional phenotypic AST is warranted to guide definitive antimicrobial therapy.

In this study, the AMR genes included in the BCID2 panel demonstrated generally high phenotypic predictive performance. In particular, the specificity, i.e., the probability that an AMR gene would not be detected in phenotypically susceptible isolates, exceeded 90% for most targets. However, the sensitivity, i.e., the ability to detect AMR genes in phenotypically resistant strains, was lower for certain targets such as mecA/C in methicillin-resistant CNS and CTX-M in ESBL-producing *Enterobacterales*. This represents a key limitation of BCID2, and it does not cover all resistance mechanisms associated with methicillin-resistant CNS or ESBL-producing organisms. In contrast, the panel demonstrated 100% accuracy in predicting phenotypic resistance for methicillin-resistant SAU and carbapenemase-producing *Enterobacterales*. These results indicate the importance of interpreting BCID2 results in the context of local epidemiology and resistance patterns covered by the test.

Analysis of the turnaround times for rapid AST revealed that the shortest times were observed in BCID2, QdRAST, and dPhoenix, respectively. The estimated total turnaround times from blood culture positivity to the result reporting is approximately 10 h for the BCID2, 15 h for QdRAST, and 22–24 h for dPhoenix using the median time from blood culture positivity to preparation initiation, which is approximately 8 h and 30 min for the BCID2, as a standardized time for all rapid AST methods. The time from blood culture positivity to preparation initiation for BCID2 was significantly longer during off-duty hours, approximately 10 h and 19 min, compared to 4 h and 19 min during on-duty hours, indicating a difference of approximately 6 h. Importantly, the time from blood culture positivity to preparation initiation accounted for >45% of the overall turnaround time in all three rapid AST methods. This result indicates the substantial effect of delays in initiating test preparation, particularly in laboratories that do not operate 24/7.

This study revealed that all rapid AST methods significantly reduced the turnaround time compared to conventional AST, even in a microbiology laboratory that does not operate 24/7 and performs Gram staining only twice daily. Notably, both the BCID2 and QdRAST were capable of providing AST results within 24 h of blood culture positivity. However, a substantial portion of the total turnaround time was attributed to the delay before the preparation initiation; thus, laboratories operating 24 h a day, 7 days a week, could achieve considerably faster turnaround times.

Although this study demonstrated that rapid AST methods significantly shortened the turnaround time compared to conventional methods, the clinical implications of this improvement warrant further consideration. Previous randomized controlled trials investigating various rapid AST platforms for BSIs have consistently shown that earlier availability of susceptibility results facilitates timely optimization of antimicrobial therapy [52]. However, many of these studies did not demonstrate a corresponding improvement in major clinical outcomes such as mortality or length of hospital stay. These findings highlight that the clinical benefit of rapid diagnostics is not solely dependent on the availability of results but rather on the timely and appropriate action taken in response. Therefore, integration with antimicrobial stewardship programs, diagnostics stewardship initiatives, and clinical decision support systems is essential to translate rapid test results into meaningful improvements in patient care.

This study has several limitations. First, the number of polymicrobial specimens and carbapenemase-producing *Enterobacterales* was limited, which restricted the ability to fully assess the performance of each rapid identification and AST method. Further validation using well-characterized isolates or larger-scale clinical studies would help address this limitation. Second, considering the aim of this study to compare the performance of rapid AST methods with conventional AST as used in routine clinical laboratories, discrepancies determined during testing were not further confirmed using additional reference methods. Hence, the study was limited in its ability to precisely define the performance specifications of each rapid AST platform. Third, the preparation of the three rapid AST methods could not be initiated simultaneously due to staffing and equipment constraints during the study period. Therefore, turnaround times were analyzed separately as the time from blood culture positivity to preparation initiation and the time from preparation initiation to result reporting. Fourth, weekend samples were excluded because rapid identification and AST could not be performed on weekends in our laboratory. Inclusion of these samples could have introduced bias by allowing conventional methods to be initiated earlier than rapid methods under such conditions. Fifth, as this was a performance comparison study using residual clinical specimens, we were not able to explore in depth the actual clinical effectiveness of each rapid AST method in real-world practice.

Despite its limitations, this study showed that each rapid identification and AST method offers distinct advantages and challenges. Notably, all tested platforms significantly reduced the time to bloodstream infection diagnosis, even in laboratories without 24/7 operation. These findings provide real-world evidence to guide the selection and implementation of rapid diagnostics in resource-limited settings. By highlighting the performance differences among platforms, this study supports diagnostic stewardship strategies that balance accuracy, speed, and laboratory workflow constraints.

## Figures and Tables

**Table 1:** Correct identification rates of two rapid identification tests (monomicrobial isolates).

Level	Gram-Positive Cocci (*n* = 103)			Gram-Negative Bacilli (*n* = 108)		
	SepsiTyper Kit (%)	Fimarray BCID2 Panel (%)	*p*-Value	SepsiTyper Kit (%)	Fimarray BCID2 Panel (%)	*p*-Value
Species	91 (88.35)	66 (64.08)	<0.0005	104 (96.30)	96 (88.89)	0.0269
Genus	101 (98.06)	96 (93.20)	0.1824	105 (97.22)	97 (89.81)	0.0269

**Table 2:** Correct identification rates of two rapid identification tests (polymicrobial isolates).

Level	Gram-Positive Cocci (*n* = 10)			Gram-Negative Bacilli (*n* = 3)		
	SepsiTyper Kit (%)	Fimarray BCID2 Panel (%)	*p*-Value	SepsiTyper Kit (%)	Fimarray BCID2 Panel (%)	*p*-Value
Complete	2 (20)	4 (40)	NA	0 (0)	2 (66.67)	NA
Partial	10 (100)	10 (100)	NA	3 (100)	3 (100)	NA

Abbreviations: NA, not applicable.

**Table 3:** Turnaround time for rapid identification tests.

Method	Gram-Positive Cocci	Gram-Negative Bacilli
	Median, Min–Max (Minutes)	Median, Min–Max (Minutes)
SepsiTyper kit	596, 160–2143	669, 150–1457
Fimarray BCID2 panel	599, 174–2327	573, 64–1566
Gram stain	432, 58–1269	408, 77–1047
Conventional method	3223, 1131–19,006	3261, 2156–6159

**Table 4:** Difference in turnaround time between rapid identification and conventional methods.

Method	Gram-Positive Cocci		Gram-Negative Bacilli	
	Median Difference, Min–Max (Minutes)	*p*-Value	Median Difference, Min–Max (Minutes)	*p*-Value
SepsiTyper kit vs. Filmarray BCID2 panel	59, 2–1391	0.3036	73, 1–1137	0.7982
SepsiTyper kit vs. Conventional method	2614, 0–18,703	<0.0005	2612, 1024–5537	<0.0005
Filmarray BCID2 panel vs. Conventional method	2627, 76–18,766	<0.0005	2607, 1002–5713	<0.0005

**Table 5:** Performance of the FilmArray BCID2 panel in predicting antimicrobial susceptibility.

AMR Gene	Microorganisms	Antimicrobial Agents	Susceptible (No.)	Resistant (No.)	Sensitivity (%)	Specificity (%)
mecA/C and MREJ	SAU	Oxacillin	13	5	5/5 (100)	13/13 (100)
mecA/C	CNS	Oxacillin	11	16	4/16 (25)	10/11 (90.91)
vanA/B	Enterococcus	Vancomycin	15	5	5/5 (100)	15/15 (100)
CTX-M	Enterobacterales	Cefepime	56	27	22/27 (81.48)	54/56 (96.43)
CTX-M	Enterobacterales	ESBL	46	25	21/25 (84)	43/46 (93.48)
KPC	Enterobacterales	Meropenem	81	2	2/2 (100)	81/81 (100)

Abbreviations: AMR gene, antimicrobial resistance gene; SAU, *Staphylococcus aureus*; CNS, coagulase-negative *Staphylococcus*.

**Table 6:** Turnaround time for rapid antimicrobial susceptibility tests.

Method	Time from Culture Positivity to Preparation Start		Time from Preparation Start to Result Reporting		Turnaround Time	
	Gram-Positive Cocci	Gram-Negative Bacilli	Gram-Positive Cocci	Gram-Negative Bacilli	Gram-Positive Cocci	Gram-Negative Bacilli
	Median, Min–Max (Minutes)	Median, Min–Max (Minutes)	Median, Min–Max (Minutes)	Median, Min–Max (Minutes)	Median, Min–Max (Minutes)	Median, Min–Max (Minutes)
Direct AST using BD Phoenix M50 system	767, 114–2102	734, 114–1424	936.5, 634–1106	837, 627–1148	1704, 896–3009	1609, 827–2411
QuantaMatrix dRAST (1.5.1 version)	712, 129–1395	711, 118–1444	405, 283–436	403, 278–436	1116.5, 529–1797	1111.5, 442–1846
QuantaMatrix dRAST (1.6.1 version)	712, 129–1395	711, 118–1444	404, 397–436	399, 278–436	1116.5, 529–1797	1100.5, 406–1846
Filmarray BCID2 panel	509, 125–1974	532, 94–1479	91, 39–106	91, 49–149	598, 216–2059	640, 184–1566
Conventional method	NA	NA	NA	NA	3227, 2476–5004	3283, 2235–6159

Abbreviations: NA, not applicable.

## Data Availability

The datasets used and analyzed during the current study are available from the corresponding author upon reasonable request.

## Supplementary Materials

The following supporting information can be downloaded at https://www.mdpi.com/article/10.3390/microorganisms13051041/s1. Table S1: Analysis of reasons for species-level incorrect identification (monomicrobial isolates); Table S2: Analysis of reasons for complete-level incorrect identification (polymicrobial isolates); Table S3: Accuracy of Two Rapid Antimicrobial Susceptibility Tests Compared to Conventional Antimicrobial Susceptibility Testing Results (QuantaMatrix dRAST 1.5.1 version); Table S4: Accuracy of Two Rapid Antimicrobial Susceptibility Tests Compared to Conventional Antimicrobial Susceptibility Testing Results (QuantaMatrix dRAST 1.6.1 version); Table S5: Difference in turnaround time between rapid antimicrobial susceptibility tests and conventional methods; Figure S1: Time from blood culture positivity to preparation start for Filmarray BCID2, stratified by on-duty and off-duty hours (*p* < 0.0005).

## Abbreviations

The following abbreviations are used in this manuscript:

AMR	antimicrobial resistance
AST	antimicrobial susceptibility testing
BCID2	blood culture identification 2
BSI	bloodstream infection
CA	categorical agreement
CI	confidence interval
CLSI	Clinical and Laboratory Standards Institute
CNS	coagulase negative staphylococci
dPhoenix	BD Phoenix M50
EA	essential agreement
ESBL	extended-spectrum beta-lactamases
ID	identification
MALDI-TOF MS	matrix-assisted laser desorption ionization–time-of-flight mass spectrometry
mE	minor error
ME	major error
MIC	minimum inhibitory concentration
QdRAST	QuantaMatrix direct and rapid AST
SAU	*Staphylococcus aureus*
VME	very major error
