# Supplementary material for: Comparative Assessment of Rapid Identification and Antimicrobial Susceptibility Testing Methods for Bloodstream Infections in a Non-24/7 Clinical Microbiology Laboratory
Source: Microorganisms. 2025 Apr 30;13(5):1041. doi: 10.3390/microorganisms13051041 (PMC12113830; doi:10.3390/microorganisms13051041)
Supplement: Supplementary file 1 [file microorganisms-13-01041-s001.zip › Supplementary figures_revised_20250415.pptx]

## Slide 1
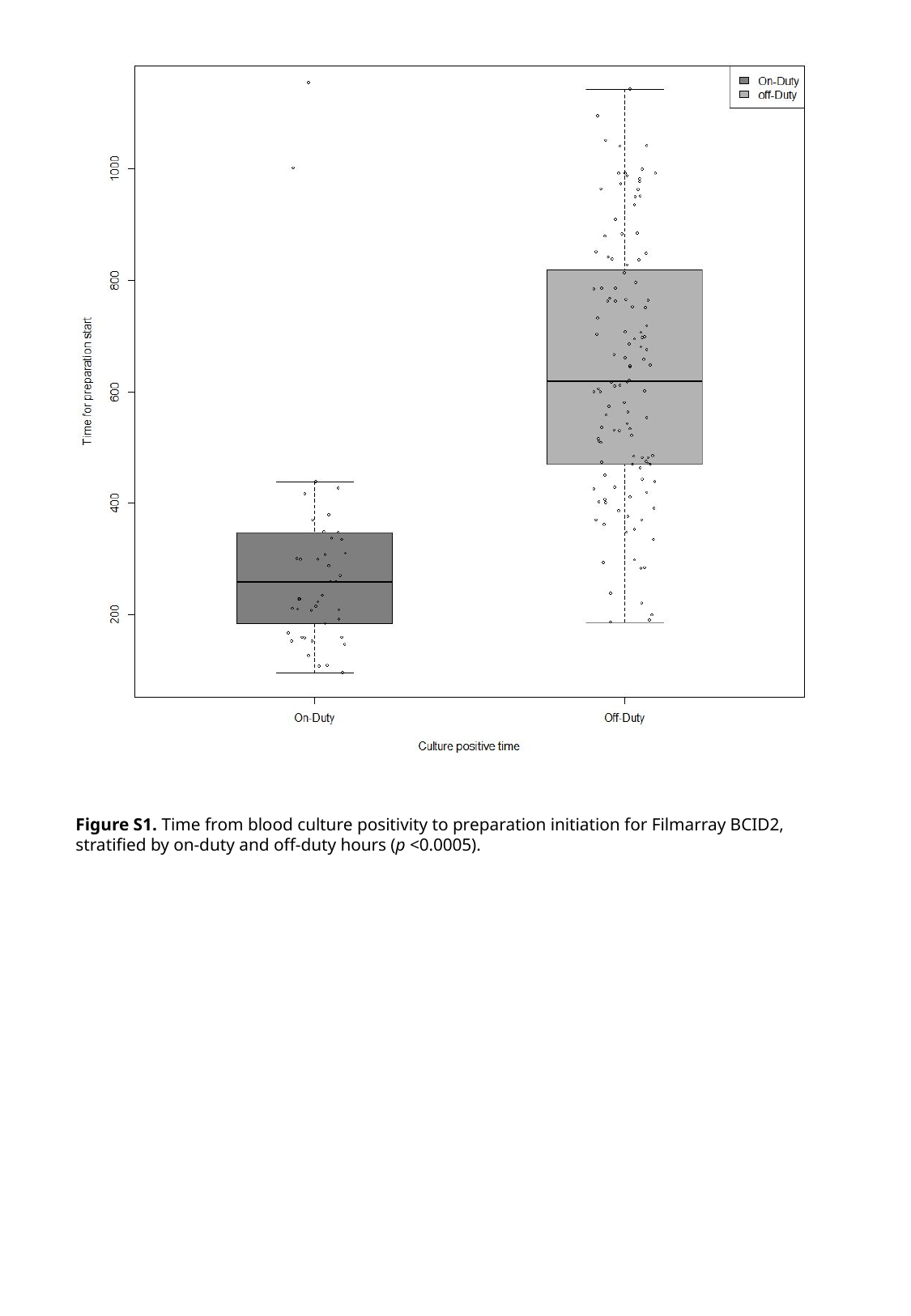

Figure S1. Time from blood culture positivity to preparation initiation for Filmarray BCID2, stratified by on-duty and off-duty hours (p <0.0005).
